# Bibliographical Notices

**Published:** 1857-01

**Authors:** 


### EDITORIAL DEPARTMENT.

### BIBLIOGRAPHICAL NOTICES.

***Geology of the Pacific and other regions visited by the U. S. Exploring Expedition under C. Wilkes, U. S. N., in the years*** 1838—1842. By James D. Dana, Geologist of the Expedition.

This report consists of a quarto volume of text of 750 pages, illustrated by several maps and numerous wood cuts, and a folio atlas of 21 plates. It treats of the structure, growth and distribution of coral reefs and islands; of the geology of the Sandwich Islands; the Society Islands; the Feejees; the Navigators; of the phenomena of volcanic action; changes of level in the Pacific, and origin of the general features of the Globe; of the geology of New Zealand, Chili, Peru and Fuega; and of a part of Oregon and California.

The folio atlas contains figures of fossils of the coal and inferior formations of New South Wales, and of the tertiary rocks of Oregon.

Only 200 copies of this government report have hitherto been printed. The author proposes to have 250 copies published for the benefit of those who are interested in the subjects. The copies will be furnished to subscribers for $12 00, the text bound in cloth, the payment to be made on delivery. A copy was recently sold in New York city for $40 00.

Should the subscription list reach 500, the edition would be increased accordingly, and the price reduced to $10 00. The work, if undertaken, will be ready for delivery in the course of the present year.

Any person desiring one or more copies is requested to send his name, and those of any others who may be induced to subscribe for the work, by an early mail, to the author, at New Haven, Conn.

We cordially commend this enterprise to our readers as one eminently deserving the encouragement of all who desire the progress of science in our country. The book has already achieved for itself a well deserved fame, and the price at which it is offered is extremely moderate.

---

*Arctic Explorations:* the Second Grinnell Exploration in search of Sir John Franklin, 1853, '54, '55. By Elisha Kent Kane, M. D., U. S. N., 2 vols. 8vo., pp. 464, 467; Childs & Peterson: Philadelphia, 1856.

This work is already so favorably and extensively known that an announcement of it, by us, will extend but very little, if at all, a knowledge of the publication. The protracted absence of the courageous and persevering adventurers, awakened in the breast of almost every American as well as European, the intensest anxiety for their safety, and many began to entertain serious fears that Dr. Kane and his fearless little band had shared the sad fate of Sir John Franklin. Every one recollects too, the universal ecstacy of joy, with which the first intelligence of the safety of the expedition was received.

The narrative of the long and intense sufferings and almost miraculous preservation of this courageous little band, is thrillingly interesting. No one after commencing its perusal can lay the book aside until it is finished. The Arctic explorations of Dr. Kane have resulted in a vast increase of geographical knowledge, and establish, we think, beyond doubt, the existence of the long conjectured "Open Polar Sea."

The book is illustrated by over three hundred finely executed engravings, and many of them on steel. It is altogether beautifully gotten up, printed on fine paper, and with large type, reflecting the highest credit upon the enterprising publishers.

---

*Painless Tooth Extraction*, without Chloroform; with Observations on Local Anæsthesia by Congelation in General Surgery. By Walter Blundell. 8vo., Churchill, London.

We take pleasure in acknowledging the receipt of the above work, and would have done so in the last number of this journal, had not the work in question been misplaced. As, however, the profession are extremely familiar with the important discovery, treated by Mr. Blundell, through Mr. Quinton's book, and other publications in the numerous medical and dental periodicals, it will not be necessary to bestow upon this volume, and at this late day, other than of general commendation.

In regard to the practice of painless tooth-extraction we are rather inclined to the opinion, that dentists will adhere to their old painful method, except, indeed, in cases of an extraordinary character, in which congelation is the *safest* anæsthetic agent which can be used. The inconvenience attending the frequent manipulation of ice and salt, will always prove a serious obstacle to its general use.

